# How to improve research capacity strengthening efforts: learning from the monitoring and evaluation of four research consortia in Africa

**DOI:** 10.1186/s12961-023-01056-9

**Published:** 2023-10-25

**Authors:** Victoria O. Kasprowicz, Caroline Jeffery, Dorcas Mbuvi, Victoria Bukirwa, Karim Ouattara, Florence Kirimi, Kathrin Heitz-Tokpa, Mary Gorrethy, Denis Chopera, Damalie Nakanjako, Bassirou Bonfoh, Alison Elliott, Samson Kinyanjui, Imelda Bates, Thumbi Ndung’u

**Affiliations:** 1 Africa Health Research Institute, KwaZulu-Natal, South Africa; 2 HIV Pathogenesis Programme, The Doris Duke Medical Research Institute, University of KwaZulu-Natal, Durban, South Africa; 3 Centre for Capacity Research, Liverpool School of Tropical Medicine, Liverpool, United Kingdom; 4 Institute of Infection, Veterinary and Ecological Sciences, University of Liverpool, Liverpool, United Kingdom; 5 KEMRI Wellcome Trust Research Programme, Kilifi, Kenya; 6 Strathmore University Business School, Nairobi, Kenya; 7 Department of Medicine, School of Medicine, Makerere University College of Health Sciences, Kampala, Uganda; 8 Centre Suisse de Recherches Scientifiques en Cote d’Ivoire, Abidjan, Lagunes Ivory Coast; 9 Catholic Relief Services, Nairobi, Kenya; 10 Uganda Virus Research Institute, Wakiso, Entebbe, Uganda; 11 London School of Hygiene & Tropical Medicine, London, United Kingdom; 12 Nuffield Department of Medicine, Centre for Tropical Medicine and Global Health, University of Oxford, Oxford, United Kingdom; 13 Department of Biochemistry, Pwani University, Kilifi, Kenya; 14 Ragon Institute of MGH, MIT and Harvard University, Cambridge, MA United States of America; 15 Division of Infection and Immunity, University College London, London, United Kingdom

## Abstract

Recent efforts to shift the control and leadership of health research on African issues to Africa have led to increased investments for scientific research capacity strengthening (RCS) on the continent and a greater demand for accountability, value for money and demonstration of return on investment. There is limited literature on monitoring and evaluation (M&E) of RCS systems and there is a clear need to further explore whether the M&E frameworks and approaches that are currently used are fit for purpose. The M&E approaches taken by four African RCS consortia funded under the Developing Excellence in Leadership, Training and Science in Africa (DELTAS) I initiative were assessed using several methods, including a framework comparison of the M&E approaches, semi-structured interviews and facilitated discussion sessions. The findings revealed a wide range in the number of indicators used in the M&E plans of individual consortium, which were uniformly quantitative and at the output and outcome levels. Consortia revealed that additional information could have been captured to better evaluate the success of activities and measure the ripple effects of their efforts. While it is beneficial for RCS consortia to develop and implement their own M&E plans, this could be strengthened by routine engagement with funders/programme managers to further align efforts. It is also important for M&E plans to consider qualitative data capture for assessment of RCS efforts. Efforts could be further enhanced by supporting platforms for cross-consortia sharing, particularly when trying to assess more complex effects. Consortia should make sure that processes for developmental evaluation, and capturing and using the associated learning, are in place. Sharing the learning associated with M&E of RCS efforts is vital to improve future efforts. Investing and improving this aspect of RCS will help ensure tracking of progress and impact of future efforts, and ensure accountability and the return on investment. The findings are also likely applicable well beyond health research.

## Background

Strong national capacity for health research is essential for addressing national health challenges [1] but is limited in many low- and middle- income countries (LMICs) [2, 3]. For example, low-income countries received only 0.2% of 69 420 biomedical grants listed on the World RePORT platform for the year 2016 [3], and countries belonging to the WHO Africa Region produced only 1.3% of global health research publications in 2014 [4], despite accounting for 14% of the global population. The United Kingdom government and Wellcome Trust alone spent £873 million between 2016 and 2021 on dedicated research capacity strengthening initiatives in LMICs, with a further £1.2 billion expended on research activities with a capacity strengthening component [5]; most of this spend was on health research capacity strengthening (RCS).

Recent efforts to shift the strategic leadership and operational control of health research on African issues to Africa have led to increased investments for scientific RCS on the continent. In turn, this has also led to a greater demand for accountability and demonstration of return on investment on scientific research capacity and health. However, there are many challenges related to measuring the impact of capacity building interventions [6]. The evidence base to inform health RCS intervention design, implementation and evaluation is poorly developed with few original research studies [7]. Robust evaluations of  health RCS interventions and standardized metrics for reliable outcome and impact data are scarce [7, 8]. As a result, substantial funds continue to be invested in health RCS initiatives globally in the absence of clear evidence to inform decision-making.

A mixture of evaluation approaches, each with merit and demerits, have been adopted, ranging from funder-commissioned end-of-programme external evaluation and funder-driven periodic internal monitoring and evaluation (M&E) reporting to programme self-initiated monitoring for real-time learning and improvements [9, 10]. Previous reports have highlighted that most M&E frameworks are primarily oriented towards meeting funders’ requirements instead of helping programmes gain a better understanding of their processes and the potential impact of their efforts [11]. However, there is limited literature on M&E of scientific capacity strengthening programmes in general, as well as on the associated learning and best practices. Several reports describing different programmes and proposed frameworks exist, but few have focussed on actual evaluations. In addition, there are limited reports on grantee experiential learning of monitoring and evaluating their own programmes. Overall, this impacts the field of M&E of health RCS initiatives by limiting the evidence on the effectiveness of such programmes [6]. It also impedes comparison of different programmes and approaches. Several efforts have been taken to support self-monitoring and evaluation of health RCS programmes, such as providing guidelines and support to develop M&E framework and indicators [12–14]. However, to date there is still no consensus on the best evaluation metrics for RCS [12]. A recent review of RCS indicators in peer-reviewed and grey literature revealed that many indicators are of low quality and are not Specific, Measurable, Achievable, Relevant and Time-bound (SMART) [12, 13]. As such, there is need for more studies to assess whether the M&E frameworks and approaches that are currently employed by RCS programmes are fit for purpose in a rapidly evolving research ecosystem.

In this paper, the study team examined and reported on the M&E experiences among four African-led RCS consortia that were part of the DELTAS Africa I Initiative (launched by Wellcome in 2015) with the aim of improving future RCS M&E efforts. Each consortia had been in operation for at least 5 years. The impetus for this study was the realization that the consortia could be doing more to share their data and learning acquired through their M&E activities for the benefit of others and to help demonstrate consortium impact. It was felt that certain features, particularly qualitative or complex intersecting aspects of consortium activities, were not optimally captured by the M&E frameworks in place. Additionally, it was felt that it would be beneficial to discuss, share with and learn from other consortia, particularly aspects that could be optimized or changed going forward. By comparing M&E frameworks adopted by the consortia, examining the influence of funders’ reporting requirements on the frameworks and documenting the experiences of implementing the frameworks, the team aimed to generate learnings that could be shared with others. In short, the study team wished to identify the most effective and suitable approaches for M&E RCS efforts to help improve the tracking of progress, success and impact of future RCS efforts and ensure accountability and the return on investment.

## Methods

### RCS programme details and participating study consortia

**Fig. 1:**
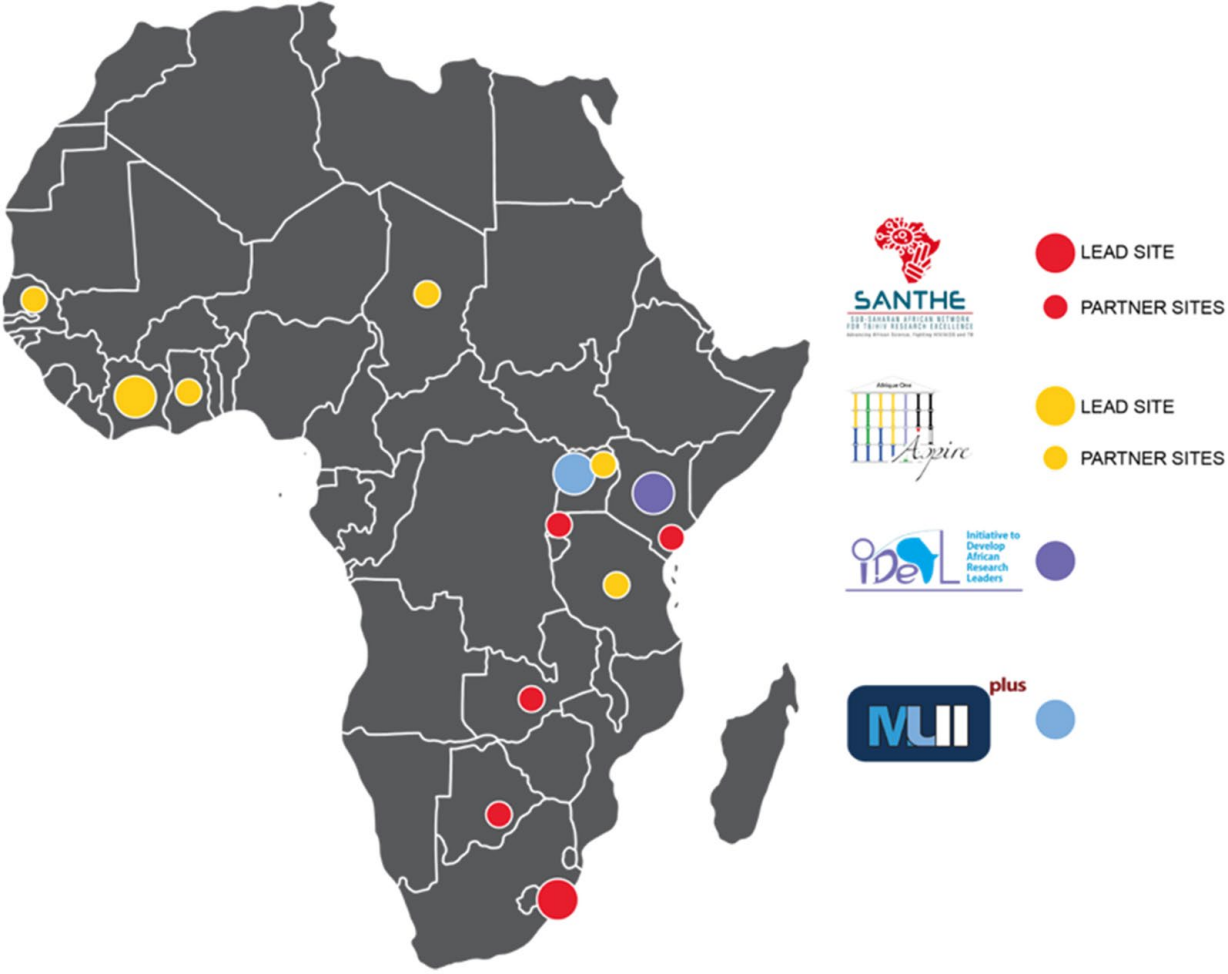
Participating consortia and locations within Africa (2015–2020)

The DELTAS Africa I Initiative initially supported 11 research consortia across Africa for a period of 5 years. A programme theory of change (ToC) was developed (https://www.lucidchart.com/documents/view/1ac12fc3-09f3-4451-8f8d-a910a928dd71/0_0). Each awardee (we will refer to these as the ‘consortia’) was expected to focus on the four key strategic areas: 1. scientific quality, 2. research training, 3. scientific citizenship, and 4. research management and environment. DELTAS Africa allowed each consortium to develop their own ToC (aligned to the programme ToC) and be responsible for their own M&E plans and processes. They also funded a DELTAS Learning Research Programme (LRP) (https://www.lstmed.ac.uk/research/centres-and-units/centre-for-capacity-research/deltas-–-learning-research-programme) to provide insight and learning on selected topics that were important to the overall initiative. The submission of a formal M&E plan was a requirement from each consortium, with the design based on their area of research, needs and the programme theory of change. The DELTAS Africa leadership provided guidance and support to the consortia during the process of M&E plan development. This support included guidance documents, templates and information sharing. DELTAS Africa required annual reporting as a grant condition, with a formal reporting template (which evolved significantly over the first couple of years) and common indicators (Table 1) provided for the annual report. The four DELTAS Africa consortia that took part in this study are the Sub-Saharan African Network for TB/HIV Research Excellence (SANTHE) (https://www.santheafrica.org), the Initiative to Develop African Research Leaders (IDeAL) (https://ideal.kemri-wellcome.org), the Makerere University-Uganda Virus Research Institute (UVRI) Centre of Excellence for Infection & Immunity Research and Training (MUII-plus) (http://www.muii.org.ug) and the African Science Partnership for Intervention Research Excellence (Afrique One-ASPIRE) (http://afriqueoneaspire.org) (Fig. 1). IDeAL has a multidisciplinary and multi-disease focus, MUII-plus has a focus on immunology of infectious disease, SANTHE has a focus on HIV and TB, and Afrique One-ASPIRE has a focus on One Health. IDeAL, MUII-plus and SANTHE are based in Anglophone countries, whereas Afrique One-ASPIRE is based in a Francophone country. Two consortia were based across multiple institutions while the other two were based at a single site. Earlier versions of three of the consortia were present from 2008/2009 as part of the African Institutions Initiative, but the SANTHE consortia officially started in 2015 with the DELTAS Africa award. Each of the RCS consortia varied in their approach, with diverse interventions being utilized. However, all the consortia engaged in research training at different levels (including undergraduate and graduate internship, Masters, PhD and postdoctoral training) which was embedded within the research activities of the consortia partner and collaborating institutions.

**Table 1:**
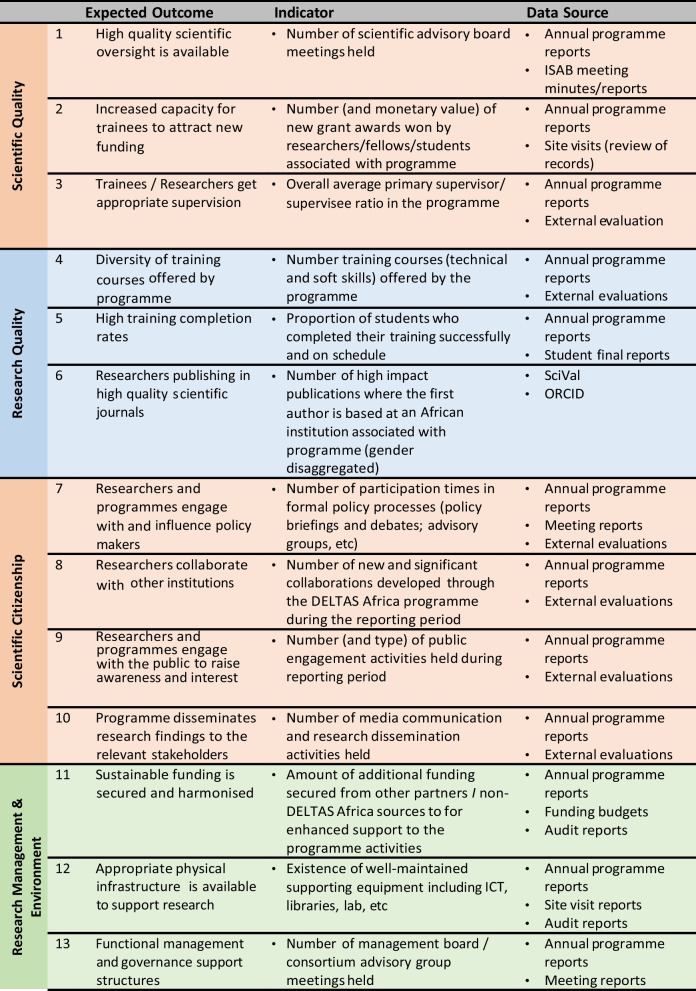
Indicators required for the DELTAS Africa Annual Reporting; included with permission from the Science for Africa Foundation

**Fig. 2:**
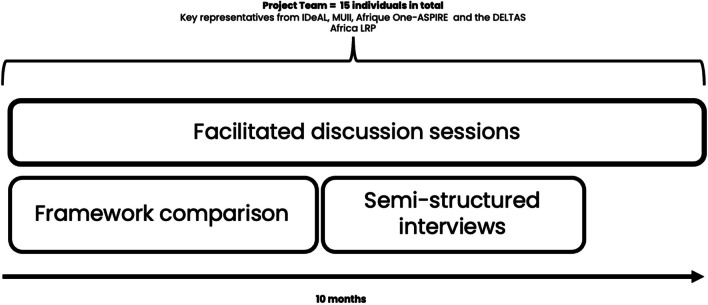
Illustration of the approach taken

Three approaches were adopted to document, compare and contrast the M&E experiences of the four consortia: 1. framework comparison of the M&E approaches, 2. semi-structured interviews of representatives from IDeAL, MUII and Afrique One-ASPIRE which were performed by the DELTAS LRP team members and 3. facilitation of meetings across the four consortia. The research team consisted of 15 individuals in total; 13 were representatives of the different consortia, typically, the director/co-director, consortium manager and M&E lead. The two additional team members were representatives from the DELTAS Africa LRP who were included to help facilitate discussions since they had extensive experience of RCS evaluations in different health and non-health contexts across Africa [15]. Our approach is displayed in Fig. 2.

### Consortia’s monitoring and evaluation plans: constructing a comparison framework

The final M&E plans were collected from each of the four consortia. These were used as a basis to design a nine-column framework (in Microsoft Excel) to contrast the approaches taken. Columns included indicator level (i.e. individual, institution, societal); DELTAS Africa Theory of Change strategic areas (i.e. scientific quality, research leadership, scientific citizenship and research management, culture and infrastructure); capacity building focal area (i.e. career development, courses and training, scientific and academic quality, knowledge exchange and collaborations, knowledge dissemination, research support services and supervision and mentorship); capacity building activity associated with the indicator; nature of the indicator (i.e. qualitative versus quantitative); indicator type (i.e. output versus outcome versus impact); expected outputs/outcomes/impact; data capture information; and additional comments. Authors, who were independent of the consortia and responsible for the DELTAS LRP, initially populated this framework using the M&E plans provided by each consortia. Each consortium then checked and amended their consortium’s sections in the framework. Each consortium identified any indicators that were in the original plan that were not actually used in practice. Indicators that were used but were not included in the original plans were added to the framework. Once the framework was complete and checked, key data were then extracted by one research team member and presented in a table summarizing the indicators and their reason for inclusion in the M&E plans (Table 2). This table was independently verified by representatives from each consortium. The data from the interviews and from the framework were discussed in virtual team meetings [due to the coronavirus disease 2019 (COVID-19) pandemic] (see below). As the team reflected on the indicators that they had used in their programmes, they were able to identify some topic areas that were not included in the original M&E plans. It was felt that these new topic areas would have strengthened the M&E plans and the ability for consortia to answer crucial questions that emerged over the course of the grant implementation period. One example of this is the sustainability of RCS efforts.

**Table 2:** Summary of the type of indicators used and reason for inclusion in monitoring and evaluation plans of four DELTAS Africa consortia for the period 2015–2020

		Consortium 1	Consortium 2	Consortium 3	Consortium 4
Total number of indicators (numbers not performed in brackets)		65 (18)	29 (0)	41 (4)	87 (13)
Nature of indicators	Quantitative	53 (12)	24	41 (4)	84 (13)
	Qualitative	12 (6)	5	0	3
Funder-defined focus areas	Scientific quality	17 (3)	6	12	30(4)
	Research leadership	25 (3)	9	12 (1)	22 (4)
	Research management, culture and infrastructure	9 (4)	7	7 (1)	7
	Scientific citizenship	13 (8)	7	10 (2)	28 (5)
Study-defined headings	Career development	19 (4)	4	7(1)	9 (1)
	Courses and training (skills development)	11	4	11(1)	19 (4)
	Knowledge dissemination or citizenship	11 (3)	3	7 (1)	21 (4)
	Knowledge exchange and collaborations	13 (8)	5	79	12(3)
	Research support	5 (3)	7	5 (1)	5
	Scientific/academic quality	3	6	4	13
	Supervision and mentorship	2	0	0	8 (1)
Indicator level	Individual	23 (3)	16	12 (1)	39 (8)
	Institution	36 (11)	9	21 (2)	21
	Societal	5 (4)	4	8 (1)	27(5)
Indicator type	Output	32 (9)	17	22 (2)	71 (11)
	Outcome	33 (9)	11	19 (2)	16 (2)
	Impact	0	1	0	0
Nature of indicators	Quantitative	53 (12)	24	41 (4)	84 (13)
	Qualitative	12 (6)	5	0	3
Reason for inclusion of indicator	Reporting to DELTAS Africa programme	1	0	11	23
	Individual consortium tracking/learning	42 (18)	16	8 (2)	10 (10)
	Both	22	13	22 (2)	54 (3)

Numbers in brackets indicate indicators listed in plans but not actually used

#### Interviews

The purpose of the interviews were to identify challenges, strategies and learning for RCS M&E to find out what did or did not work well and why. From the participating consortia, we invited consortia directors, consortia managers and M&E staff to participate in one-on-one interviews. Individuals were selected for an interview on the basis of the need to explore a diversity of views from people with a range of roles in the consortia, especially those responsible for management and M&E aspects in the consortia. The topic guide was developed from the analysis of the M&E framework, and covered eight questions all related to capacity strengthening: their initial vision, what success would look like, unanticipated achievements, evidence for successes, suggestions for better indicators of success, what did not work so well and why, techniques for capturing data/evidence, ways of using evidence for learning, and recommendations for future programmes.

The interviews were conducted virtually by the team members from the LRP. All participants were asked to provide consent prior to the beginning of the interview. The data collected from the interviews were recorded when consent was given. Six participants were interviewed (one consortium director, three consortium managers, two M&E officers) during 2021. Written notes were written up on the same day as the interview whenever possible to minimize recall bias. Further clarification was sought from the audio recording if necessary and the recordings were destroyed after the notes from the interviews had been checked against the recordings. To safeguard participants and to allow data to be shared while maintaining the interviewees’ privacy, interview data were anonymized. Each transcript was given an anonymized ID so that it could be traced back to source (e.g. C2D, C3M, C4O, etc., indicating Consortium 2 Director; Consortium 3 Manager, Consortium 4 M&E Officer, respectively). The interview data were mapped onto a pre-designed matrix on the basis of the eight questions in the topic guide (which had been based on the M&E framework). Responses to each question were analysed separately to identify themes that were similar across the responses the those that were different. Some themes recurred across different questions. They were also compared by mapping the responses against the M&E framework, and could coincide with themes or issues identified from the M&E framework analysis and facilitate discussion meetings.

### Facilitated discussion meetings

Eight virtual team meetings took place over a period of 10 months. These meetings provided opportunities to discuss the consortia’s experiences of conducting their M&E activities, including the results of the M&E plan comparison and the interview data. The discussions also focused on the suitability of the funder M&E reporting framework to capture all aspects of the programme implementation and the challenges of aligning this with the consortia’s theory of change. The discussion sessions also reviewed results of the M&E framework comparison. The data were discussed in the virtual team meetings. As the group reflected on the indicators that they had used in their consortia M&E framework, including reasons for excluding indicator that had been outlined in the funding application but were subsequently dropped and vice versa, we were able to identify some topic areas that were not included in the original M&E plans.

### Ethical approval and consent to participate

Ethical approval and letters of support from the institutions hosting the consortia were obtained (Liverpool School of Tropical Medicine reference number protocol 18-092).

## Results

### Number, type and level of indicators used by the study consortia in their M&E plans

Table 2 summarizes the type and number of indicators listed in the M&E plans of the study consortia. The number of indicators in the consortia’s original plans ranged from 29 to 87. However, three of the consortia dropped several of the indicators during the implementation phase of the consortia. As presented in Table 2, after dropping some of the indicators, Consortium 4 retained the highest number of indicators (74). This was followed by consortium 1 (47), then consortium 3 (37). Consortium 2 had the fewest indicators with only 29 in its framework. The main reasons given for not using some of the originally planned indicators were that consortia were unsure how best to capture the required data, or decided that the indicators were not useful.

**Fig. 3:**
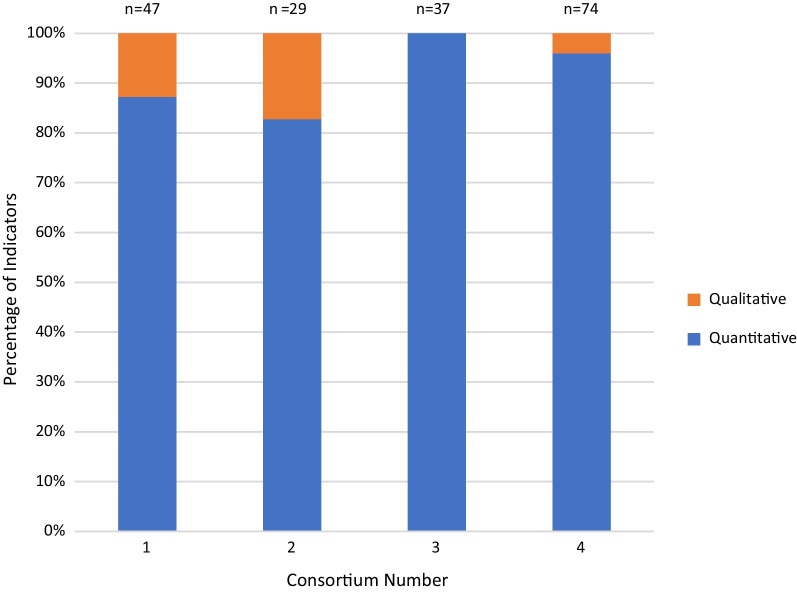
Percentage (*y*-axis) of quantitative indicators as a total of the whole number of indicators used per consortium (*x*-axis)

All 13 of the indicators provided by the DELTAS Africa programme were quantitative in nature and focused on key consortium processes, for example, number of meetings held; or key outputs, for example, number of publications (Fig. 3). This focus on quantitative indicators was present in all the M&E plans included in this comparison (Fig. 3). All of consortium 1’s indicators were quantitative in nature, while 80% or above were for the other consortia (Fig. 3).

**Fig. 4:**
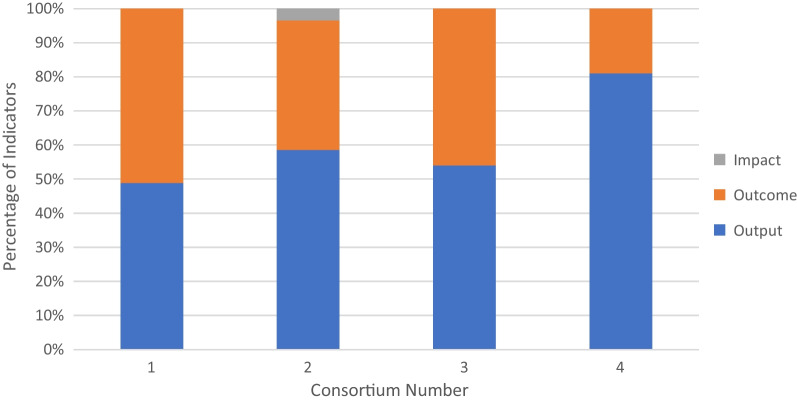
Percentage of indicators (*y*-axis) that were used by the four study consortia split by type (output versus outcome versus impact) on the *x*-axis

**Fig. 5:**
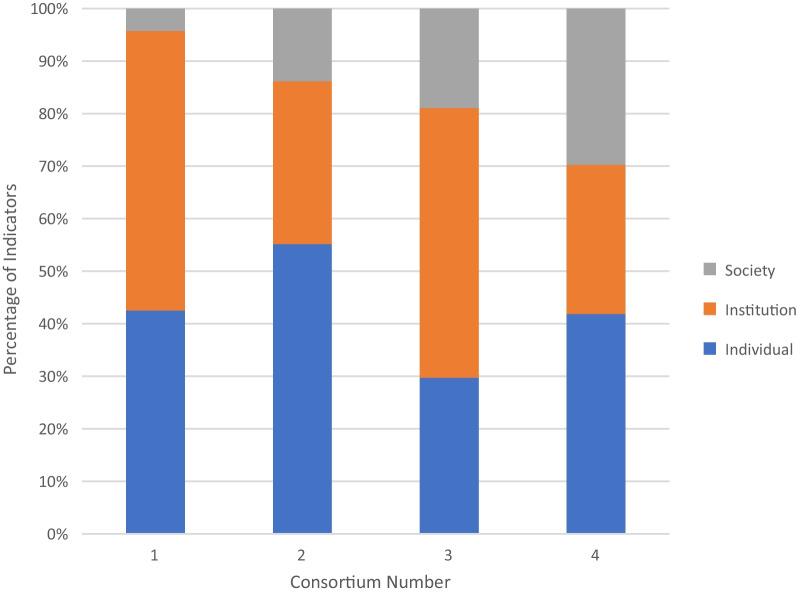
Percentage of indicators (*y*-axis) that were used by the four study consortia split by level (individual versus institution versus societal) on the *x*-axis

The type of indicators used in the M&E plans was also looked at, that is, output, outcome or impact indicator (Fig. 4), and the level of capacity building being measured, that is, measures of individual versus institutional versus societal research capacity (Fig. 5) (individual: at the level of individual scientists; institutional: at the level of an institution/organization; societal: at the level of national and regional systems) [12, 15]. Most of the indicators used were a mixture of output and outcome indicators with only consortium 2 listing one impact indicator. Consortium 4 focused mainly on output, while the other two consortia had a balance between output and outcome indicators. In general, the consortia varied considerably with regard to the scope of capacity building (whether individual, institutional or societal). The consortia differed in their indicator focus, with consortia 2 and 4 having most of their indicators targeting the ‘individual’ level and consortia 1 and 3 having the majority of their indicators targeting the ‘institution level’. All consortia had the lowest number of indicators targeted at the ‘societal’ level except for consortium 4.

### Focus areas of indicators used in M&E plans

**Fig. 6:**
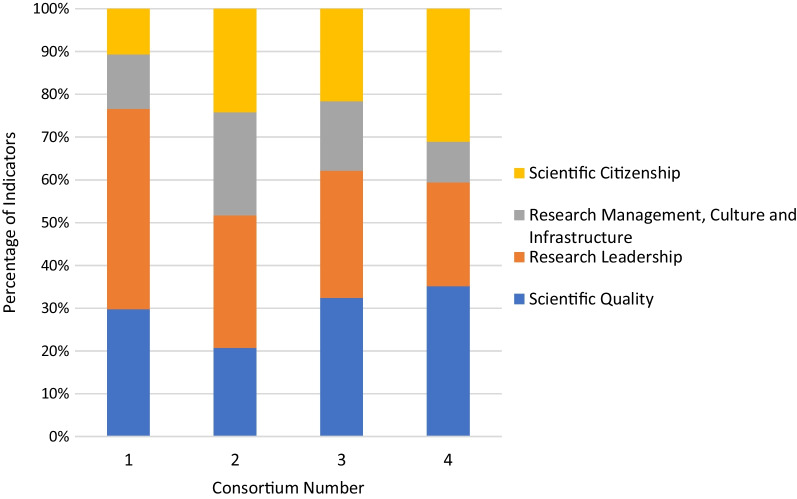
Spread of indicators used by the four study consortia over the four programme focal areas; percentage of indicators are reflected on the *y*-axis and the different consortia are represented on the *x*-axis

**Fig. 7:**
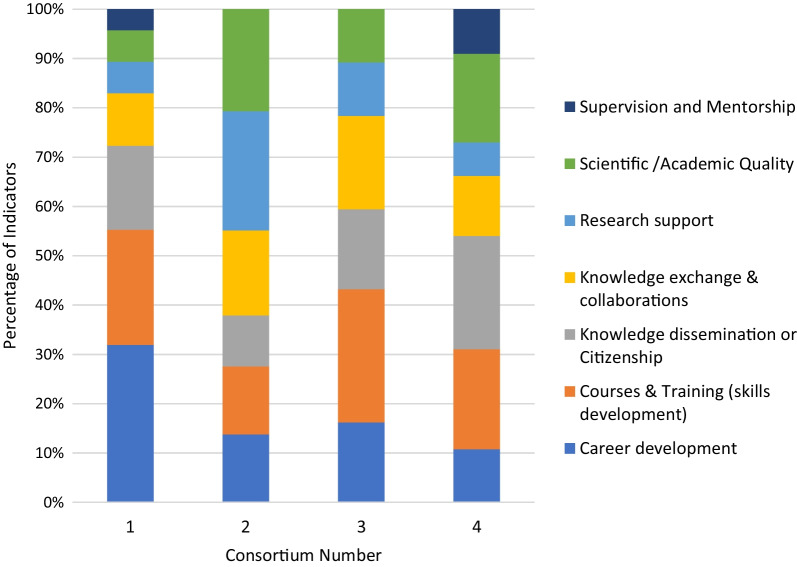
Spread of total indicators used by the four study consortia over our project-defined target areas; percentage of indicators are reflected on the *y*-axis and the different consortia are represented on the *x*-axis

The spread of the indicators used by each consortium, as split by the four programme target areas, was then examined (Fig. 6). Most of the consortia had their indicators reasonably well spread out across all four of the programme-defined target areas, particularly consortium 2. Consortium 1 was the main exception, with the majority of indicators focussing on the scientific quality and research leadership categories. Overall, totalling all the indicators across all four consortia, the greatest number of indicators were in the research leadership category, closely followed by scientific quality, with the fewest indicators in the research management category. The team then looked at the indicators split by the RCS focal areas defined as part of this study (Fig. 7). Three consortia (2, 3 and 4) had their indicators somewhat evenly distributed among the different project-defined focal areas, while consortium 1 had most of the indicators focussed on career development, courses and training. Interestingly, consortia 1 and 4 had limited indicators in the category of supervision and mentorship, while consortia 2 and 3 had no indicators in this focal area. Overall, the greatest number of indicators fell in the courses and training category, followed by the career development category. Together, it is clear that the consortia M&E efforts to date were heavily focused on areas such as training, with limited focus on more strategic outputs and outcomes.

### Reporting on progress in RCS

**Fig. 8:**
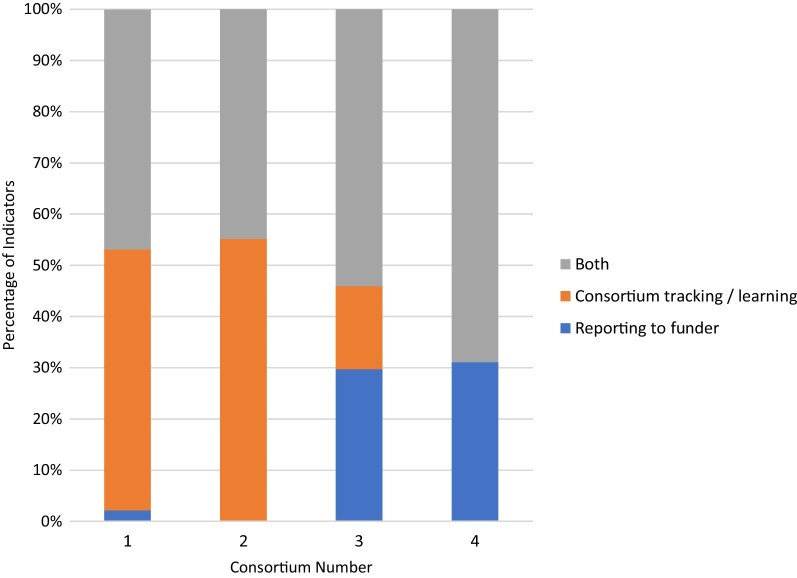
Reason for inclusion of indicators in the monitoring and evaluation plans: either reporting to the DELTAS Africa programme management for individual consortia tracking and learning, or both. Data are shown for the indicators actually used per consortia. Percentage of indicators are reflected on the *y*-axis and the different consortia are represented on the *x*-axis

The DELTAS Africa programme leaders provided an overarching ‘programme level’ theory of change (ToC). They asked each consortium to develop their own ToC aligned to the programme ToC, and also to design their own M&E plans (while providing common indicators to help with programme reporting). It was felt that this evaluation approach was generally effective at supporting consortia better understand and improve their efforts. This enabled the consortia to map their progress to the overarching programme TOC while being able to also explore other aspect of progress specific to their own needs and goals. The consortia benefitted from an evaluation model which allowed key consortia representatives to be both heavily involved in the M&E and RCS implementation. All consortia tried to merge the obligation to report to the DELTAS Africa programme with their own need to monitor their activities and document the lessons they were learning. Table 2 and Fig. 8 outline the reason for inclusion of indicators in the M&E plans. For all consortia, most indicators were included for individual consortia tracking/learning purposes. Only consortia 2 and 3 had a significant number of indicators that were included in the M&E plans only for reporting purposes (to the DELTAS Africa programme management). However, these data show that the consortia’s M&E plans were used mainly to support efforts to learn more about their own performance. Interestingly, there was some feeling expressed in the interviews that consortia should be allowed to set their own goals rather than solely rely on the programme’s theory of change. However, in the programme call and consortia applications, the instructions were for consortia to develop their own ToC that was aligned to the overarching programme ToC so they could set their own goals, but within parameters. The team are aware that the purpose of having an overall programme ToC was to accelerate learning about RCS (through comparisons and pooling of data) and to enhance the potential for impact of the whole programme.

The consensus by all four consortia was that the DELTAS Africa annual reporting template was complex and time consuming to complete and did not necessarily add significant value to their own efforts in tracking consortia’s progress. Furthermore, the template lacked the design to capture qualitative information, such as informal aspects of consortia meetings and success stories. Consortia felt that more effort needed to be dedicated to capturing and sharing qualitative data as a mechanism to better understand progress and also to eventually help demonstrate success. Each interview transcript was given a unique ID (in square brackets—see “Methods”) so it could be presented anonymously but also traced back to source.

‘We need to find improved ways to share qualitative data as well as strengthen capacity of the M&E personnel. We need diversified reporting to do this’ [C3O].

Individual interviewees would have liked to include reporting on public and community engagement and the impact of their RCS activities.

‘In the future we would like to develop success stories, with help from the communication office’ [C4O].

### Proposals to improve RCS M&E approaches

The consortia members provided suggestions for methods of demonstrating success for RCS in future programmes. This included the commonly mentioned improved use of qualitative approaches, capturing detailed aspects of the provided training, or the support of peers and mentors. Other suggestions included: measuring ‘soft’ skills (e.g. leadership), career tracking of fellows, attribution of research outputs to their institutions and contributions made to the communities (in addition to that of contributions to policy).

‘We have honorary fellows that are not members of [C3], but we support them in other ways – office space, sponsorship, etc. This has exposed them to a number of good experiences. In the end, they do mention us in their publications and successes. This has helped us grow as a family and a program. We take on fellows funded by other programs to grow collaboration’ [C3M].

Examples of some additional qualitative indictors that could be beneficial in future efforts included ‘evidence of graduates critical thinking skills’, ‘benefits of ‘peer-to-peer’ mentorship’, ‘confidence and empowerment in potential research leaders’ and ‘collaboration across diverse stakeholders’. The consortia further identified RCS areas for additional indicator development that were not included in the original M&E plans, including sustainability of consortium efforts, diversity and inclusion, impact of consortium membership on sites, quality of site–site interactions within a consortium, translating research into policy and practice, multi-disciplinarity of ongoing research projects, employability of consortium graduates, career pathways, community and public engagement, science communication and impact of publications, improving the staffing and infrastructure for research managers in institutions, networking between consortia, capacity building on M&E, cost-effectiveness of RCS activities and mutual learning with donors.

‘Supportive research infrastructure like Library, IT, labs. These need to exist or be built and they need budgeting for’ [C4O]

‘Our research is considered “multi-disciplinary” (MD). We have expertise from social sciences, economics, and health. Academia in Africa does not recognize multi-disciplinary research. However, problem-driven research is multi-disciplinary. To address this, we are considering the idea of creating a particular department for multi-disciplinary research’ [C2D].

‘Collaboration with universities was not so successful. We see research institutions and universities as very separate in Africa. The time allocated for research when working with universities is very low. The governance system of research in universities in very rigid so it is difficult to bring new ideas and innovations in terms of governance’ [C2D].

Topics that dominated much of the conversations were how to optimally: (1) measure qualitative success such as personal transformation of fellows going through the DELTAS training and the impact on their career pathways and employability; (2) measure the sustainability of our efforts; and (3) capture activities that have a complex path to impact, such as policy engagement.

‘Consortium needs to be supported in M&E and finding out lessons learned. We need recurring support from DELTAS. The M&E personnel also need guidance and capacity building on most of the activities they do because things keep changing time and again. If we [institutions from different consortia] are all funded under DELTAS, we find that when we meet and share challenges, we can plan for a way forward. There should be more to M&E than just completing the annual report at the end of the year. Networking with other M&E staff from other consortia should be done to build capacity’ [C3O].

As an example, for the last point here, there was a feeling of uncertainty as to how to measure not only impact on policy, but also the value of this. The scientific activities of the consortia cover the spectrum of science from basic to clinical to implementation. The group reflected on the fact that many basic science outputs (or indeed more implementation-focused projects) do not directly impact on policy in the lifetime of that project. Rather, these scientific findings form a vital steppingstone in the pathway of scientific discovery that will ultimately impact on policy. With regards to point 2, that is, capturing the sustainability of consortia efforts, some suggested attempts to do this could include looking at institutional strengthening as a result of RCS activities and/or looking at capturing trainee career pathways as success stories. In addition, discussion and sharing around new methods should be encouraged. One theme that emerged from our discussions was the culture of oral story telling in Africa and how this can best be harnessed to enhance our efforts, with thoughts around the best storytelling tools and applications to social media.

‘Some indicators/metrics are difficult to capture in traditional M&E, such as storytelling’; ‘African traditions need to be considered. Bringing elements into writing is not really common. The M&E system should take storytelling into account’; ‘Storytelling should be incorporated into the template. Zoom calls to discuss research between researchers should be used for evaluation’ [C2D].

In addition, in the discussions and interviews, consortia members felt that they struggled on how best to monitor their RCS impact and felt that it would be beneficial for some guidance to be provided on how to measure impact at the beginning of the project. There are examples of other programmes that do this, for example, the GCRF programme provided workshops and advice at their launch meeting. RCS takes additional time over and above that which is normally allocated to research projects. Research leaders need to be given explicit guidance about the emphasis that programme management/funders expect on RCS versus the primary research, and this should be reflected in the funders’ evaluation criteria [10].

‘We need to find way to capture impact at the end of the programme period, while being able to streamline and drop attribution versus contribution from the project. This needs to be given attention. Of course, the project has contributed capacity building and has given skills to the fellows. But when a fellow begins to improve their position and move up in the ranks, they are working with other organizations as well, so you can’t say 100% of their improvement is contributed to by their experience on the project. There needs to be a way to capture the contribution or attribution made by DELTAS. The fellows should be able to share their story as individuals. They could say how much is attributed to the DELTAS project. This would need to be captured qualitatively, through hearing their experience. I don’t think this can be captured quantitatively’ [C3O].

### Need to improve approaches beyond simple indicator definition

It was clear from the discussions that there is a need for indicators that are informative and meaningful to all those involved. This will incentivize collection of high-quality data against the indicators. In addition, efforts also need to move beyond simple indicator development to identify the best methods to measure these indicators and generate high-quality data [12]. By focussing on both appropriate metrics and approaches for both qualitative and quantitative indicators, the quality of M&E should improve. More sharing and learning from others in how they approach their data capture processes is vital to help enhance M&E efforts, for example, by sharing examples of the data capture tools that have been designed and used. There is frequently significant overlap in the information that is being captured, and hope to capture in the future and sharing best practices around approaches to data capture would have wide interest. These tools (and associated processes) are crucial to high-quality M&E, and by sharing them and what worked or did not work, they can be optimized for all consortia going forward. Through the discussions described here, further areas for sharing, learning and development have been described: (i) refining indicators to improve quality and focus, (ii) identifying appropriate means of validation for indicators, (iii) defining indicators for new scientific research capacity strengthening areas, (iv) improving the use of qualitative indicators – effective conceptualization and measurement, and (v) cost-effectiveness of M&E, including time and resources allocated and outcomes.

### Approaches to RCS learning across the study consortia

Each consortium had a different approach to generating learning related to implementation of their consortia. These approaches were informed by discussion during the consortia’s steering committees and administrative meetings and by feedback from the consortia’s fellows. Although the learning was reported by interviewees to be ad hoc, it did stimulate suggestions for better-planned RCS during the next DELTAs (or other) programme.

‘The monitoring is done by the programme manager and the learning flows from it. Then we want the evaluation to be external, yearly. We want to make an action plan after the management board, make a yearly plan to be implemented. This will make us more effective in the next phase’ [C2M].

‘We have feedback surveys, e.g., after each training. This is all done online. Every Monday, we have a training department meeting. On the first Monday of the month, we invite the student representatives at that meeting, to discuss the feedback. There is also the higher degree committee, which meets twice a year. We have a shared drive. Once a month (first Wednesday), there is a meeting with heads of scientific departments, where we discuss any issues or feedback. We can also bring issues to the executive management committee on an ad hoc basis, for example, when policies need to be revised and re-approved’ [C4M].

From our discussions it was clear that some consortia captured additional information which was not formally outlined in their own M&E plans [16], and this was one contributing factor to the difference in indicator numbers between consortia.

‘The conference organized by [C4] was attended by fellows, senior researcher or PI, where formal and informal exchange of ideas on what has worked, what hasn’t takes place. This was not captured officially, however. (…) After their training, [fellows] can come back and ask for support in preparing for interviews for scholarships. This support is captured in email conversations, calendar invites, informal conversations; there is no formal process to capture this information. Tracking progress on success in getting scholarships is the indicator’ [C4O].

This information was not only linked to evaluation for accountability but also as evaluation for learning. One clear recommendation that came from the facilitated meetings discussions and the interviews was that consortia should establish mechanisms for developmental evaluation and ongoing learning, in addition to the formal M&E frameworks (Fig. 3). This would help consortia to more effectively document their learning and share learning beyond the individual consortium.

‘You need to be transparent and flexible with the institutions. We provided information about the difficulties we faced in this project, but as a result of this, we were told we were not delivering enough. The more information you provide about the challenges in the project, the less chances you are given. We have been audited many times because we needed to know where our capacity needed to be built. You think: why are other institution’s outcomes better than ours? Is it because we are too transparent with our difficulties? The learning must be different from the evaluation. If funders put learning as a front line it will help better than just having M&E. It gives transparency and trust as you can help each other. If it is just M&E, we will just show what we want you to see. The reporting is not balanced. It is important to bring different perspectives. We need to feel that our opinions are considered. Reports tend to highlight one or two stories from larger African institutes, with less said from small institutes. Each perspective needs to be in the reports. We feel we are not represented. We prefer to be in a partnership with the funder’ [C2D].

## Discussion

The aim of this study was to generate learning that could improve future RCS M&E efforts by comparing M&E frameworks adopted by four consortia funded under a common initiative. The team examined the influence of funders’ reporting requirements on the frameworks, and documented the experiences of implementing the frameworks. Our findings revealed a wide range in the number of indicators used in the M&E plans of individual consortia. However, the indicators were uniformly quantitative and mainly at the output and outcome levels. Overall, the greatest number of indicators fell in the ‘courses and training’ category, followed by the ‘career development’ category. This may be in part due to the relative ease of capturing data which are quantitative at the individual level, and because a large number of RCS activities are targeted at developing scientists. Interestingly, in the consortia M&E plans the fewest indicators fell in the research management category, highlighting in part the relative gap in efforts focussed on this category. Research management was one of the four strategic areas of focus defined by the programme ToC. Strengthening research management is a highly effective RCS focus since it will improve the effectiveness of all research activities in an institution, yet so far it has been very neglected both in terms of activities and tracking [17, 18]. The consortia varied considerably with regards to the scope of capacity building (whether individual, institutional or societal). Guidance on the focus for RCS (i.e. individual versus institutional versus societal) and the suggested weighting was not provided by the DELTAS Africa programme. The team were unable to identify an obvious explanation for the observed differences in the M&E plans of the different consortia; for example, it did not seem related to the maturity of the consortia (i.e. whether they had worked together before the DELTAS African programme initiated). However, some differences may be related to the fact that consortia captured additional information which was not formally outlined in their M&E plans. In addition, these differences may partly reflect the different types of research and the goals of each consortium. However, the process of performing this M&E plan comparison, the accompanying interviews and the in-depth discussions that took part in the facilitated discussion sessions allowed for the consortia to reflect and outline opportunities to improve future M&E efforts. This should be of interest to all those involved in RCS. In the future it may be helpful to adopt this model across a whole programme.

Consortia M&E plans are meant to help each consortium assess whether they were on course to achieve their original RCS-defined goals, while also being a requirement for the funders for accountability. The consortia were generally appreciative of the evaluation approach taken by the DELTAS Africa initiative, providing an overarching ToC and allowing for each consortium to design their own M&E plans while providing common indicators to help with programme reporting. However, it may be advisable in the future for programme managers to make more effort to explain the rationale and purpose of their programme ToC to awardees so that they can appreciate how their projects fit within the ToC, and how together all the projects contribute to achieving impact. The reporting template used by DELTAS Africa was found to be time consuming and of no real additional benefit to individual consortia. In the future, perhaps programmes could also work with RCS consortia to routinely refine indicators and influence reporting during the funding and reporting periods of the scheme. There was some disconnect between funder tracking of the effectiveness of the overall DELTAS program (partly based on the ToC theory of change) on enhancing research capacity and the impact of the consortia themselves. One suggestion for improving future efforts would be for formal M&E to be minimized and the focus instead to be on accountability for resources, and then to allow consortia to put in place a more learning orientated/flexible type of sharing approach, particularly for qualitative information (e.g. success stories, challenges and solutions, etc.). This and other key suggestions are highlighted in Table 3. Funding to support M&E was covered through the main award to the consortia, highlighting the importance of funding support for RCS M&E efforts. This helps to ensure adequate resources are in place to facilitate high-quality processes (also highlighted the value placed on M&E within the DELTAS Africa initiative) (Table 3).

**Table 3:** Summary of key suggestions for improving future RCS efforts

	Target audience	Suggestions for improvements
1.	Funders or scheme/programme managers	Strengthen networks and provide platforms to support these efforts to enable effective sharing of approaches, experiences and learning with others involved in research capacity strengthening
2.	Funders or scheme/programme managers	Ensure there is adequate funding to support high-quality monitoring and evaluation efforts
3.	Funders or scheme/programme managers	Routinely engage with research capacity strengthening consortia to refine indicators and influence reporting; although vast majority of RCS is not done through consortia but is instead embedded within the larger research programme
4.	Consortium leaders	Provide funding support to allow continuing professional development for monitoring and evaluation consortium staff, with a particular focus on capacity strengthening challenges
5.	Consortium leaders	Look beyond the ‘simple’ identification of indicators to the processes and tools used to capture the needed data; it is vital that the approaches used are shared with other consortia to help establish best practices
6.	Consortium leaders	Identify and/or refine high-quality and useful indicators for RCS, with an increased focus on the use of qualitative indicators
7.	Consortium leaders	Ensure that timely processes for developmental evaluation and learning are in place and that learning is documented and shared to benefit others when possible

Although RCS programmes have common areas of interest and overlap, there has been very little effort to harmonize ways to measure their effectiveness. This means that many opportunities have been missed to learn from comparisons across programmes and consortia. However, a recent review of indicators used by RCS initiatives reported that 63% of the outcome indicators focussed on four main areas: research management and support; research skills and knowledge; research collaboration; and knowledge transfer [12]. As others have noted, these areas of overlap between programmes are ideal places to start to jointly define and agree on generic indicators that are SMART, helping to establish standardized health RCS evaluation metrics [12, 19]. There was a general acknowledgment that additional information could have been captured as part of the consortia M&E plans to better capture the success of RCS activities, track whether consortia were on course to achieve their RCS goals, contribute to the learning taking place and measure the ripple effects of the efforts that have been implemented. Example areas, identified in the facilitated discussion sessions, are highlighted in Table 4. Capturing the ripple effects can be complicated, costly and take time, but could also be of tremendous value in better understanding the true impact of RCS activities [20]. One approach to improving future M&E of RCS, in addition to including additional areas of our activities/efforts in M&E plans, would be to include the effective use of qualitative indicators. The strong focus observed on quantitative data, such as numbers of trainees, is limiting in terms of M&E that is useful for learning about how RCS was achieved (or not) and for future improvements [21, 22]. The inclusion of qualitative indicators in M&E plans is a key recommendation for future efforts in this arena. Through our discussions, it was clear that this lack of inclusion of qualitative indicators was due to a level of uncertainty about how best to define these indicators and capture the data required. In addition, it is more time consuming. Qualitative data collection requires a different skillset from traditional M&E, and would typically mean employing social scientists to obtain high-quality data. There should be no expectation that M&E team members obtain these specialist skills, however, there is a role for assisting them with training to better understand more about how qualitative methods work (Table 3). Support to assist qualitative data capture should be budgeted for in future programmes. It will assist in capturing more depth rather than breadth of information, while also usefully exploring more about ‘why and how’ rather than ‘how much’, and assist the learning that takes place around RCS.

**Table 4:** Additional areas identified by consortia to consider including in RCS M&E plans

Additional areas to consider for RCS indicator development
Evidence of graduates’ critical thinking skills and other skills gained through aspects of provided training
Benefits of ‘peer-to-peer’ mentorship
Confidence and empowerment in potential research leaders
Collaboration across diverse stakeholders
Soft skills gained through consortia activities
Career tracking of fellows
Contributions made to the communities beyond policy
Attribution of research outputs to their institutions
Sustainability of consortium efforts
Impact of consortium membership on site
Diversity and inclusion
Quality of site–site interactions within a consortium
Translating research into policy and practice
Multi-disciplinarity of ongoing research projects
Employability of consortium graduates
Career pathways
Community and public engagement
Science communication
Impact of publications
Improving the staffing and infrastructure for research managers in institutions
Networking between RCS consortia
Capacity building on M&E
Mutual learning with donors
Cost-effectiveness of RCS activities
Consortium growth, for example, quality of relationships, as well as systems

Future efforts could identify additional outcome and impact indicators and reflect to see whether additional indicators at the societal level (e.g. networks and collaborations, influence on policy- and decision-making) would be appropriate. The lower number of indicators targeting the societal level is not surprising, as the majority of RCS initiatives/interventions to date tend to be focussed on impact at the individual or institutional levels. Additionally, societal level achievements tend to take longer – beyond the life of the funding period – and direct attribution to activities is challenging because of dilution of multiplier effects from other initiatives and programmes. The discrepancy between the relatively short-term funding period and the challenge of being able to demonstrate true long-term impact is the reason – in part – that efforts are often focussed on short-term processes versus measuring long-term impact, and may be a contributing factor to the overall lack of impact indicators in the consortia M&E plans [13, 14]. These data reflect those in a recent article by Pulford et al. which looked at indicators used by RCS initiatives and found that 40% measured output, 59.5% measured outcome and only 0.5% measured impact [12]. Together with our findings, this suggests a general lack of focus on impact indicators in research capacity building frameworks, potentially linked to the short funding timelines for programmes and a lack of plans for long-term evaluation. Consortia instead need to work out in advance what the proxy indicators are that they can measure to show that they are on a trajectory to impact. This is a reason that each consortium should have their own ToC (linked in part to the Toc of the funders/programme), as by doing this they can establish what indicators to use. There is a general assumption that how to do this is already known, but that clearly is not the case, and additional training and sharing of approaches would be beneficial (Table 3). Funders/programmes do tend to understand the limitation in trying to measure impact, however, it is also up to the researchers/consortia to make them further aware of what is and is not reasonable and to come to an agreement as to suitable intermediate /proxy measures [18]. Linked to the concept of assessing impact is the issue of attribution versus contribution. More discussion and guidance need to take place around appropriately addressing this, as there is no formula and it becomes harder the further from the activity you look.

Overall, this study has highlighted two major but linked challenges that need to be addressed to improve M&E of RCS efforts. Firstly, identifying useful indicators (both qualitative and quantitative) in key RCS focal areas, and secondly, the actual processes of obtaining appropriate data for those indicators. It is vital that those involved in monitoring, evaluation and learning of RCS efforts engage with each other effectively to share approaches/experiences and identify best practices to advance RCS together [23, 24], and funding should be made available to support and facilitate these interactions. Key areas for sharing, learning and development include: (i) refining indicators to improve quality and focus, (ii) identifying appropriate means of validation for indicators, (iii) defining indicators for new scientific research capacity strengthening areas, (iv) improving the use of qualitative indicators – effective conceptualization and measurement, and (v) cost-effectiveness of M&E, including time and resources allocated and outcomes.

## Conclusions

It is beneficial for RCS consortia to develop and implement their own M&E plans. However, these efforts could be strengthened by routine engagement with funders/programme managers and other key stakeholders to further align efforts and assist with identification and refinement of indicators. Additional guidance or training for those involved in designing and implementing M&E plans would be beneficial to help increase qualitative data capture around RCS efforts. Specialists with qualitative research skills could help to obtain the robust/high-quality data needed to influence a change in approach to learning about how to improve RCS. M&E efforts could be further enhanced by supporting platforms and activities for cross-consortia sharing and brainstorming: to design appropriate data capture tools, develop relevant indicators and assess more complex RCS effects such as sustainability and impact. The refinement of best approaches to evaluate consortia is vital, especially as RCS efforts further develop, and investment in platforms to support these efforts and this community of practitioners should be encouraged, including online learning platforms and the use of online data capture options. Consortia should ensure that processes for learning are in place, that learning is documented and that it is shared to benefit others when possible. The recommendations and reflections should assist in strengthening M&E RCS efforts and in turn help develop a good evidence base for the effectiveness of RCS activities [6, 11, 25]. Sharing the learning associated with M&E of RCS efforts is vital to improving future efforts. Investing and improving this aspect of RCS will help ensure tracking of progress and impact of future efforts, and ensure accountability and return on investment. We anticipate that our recommendations and reflections will assist in strengthening M&E RCS efforts beyond health research, and in turn help to develop a good evidence base for the effectiveness of RCS activities in Africa and around the world.

## Data Availability

The datasets during and/or analysed during the current study available from the corresponding author on reasonable request.
